# Alterations of gut microbiota and short-chain fatty acids induced by *Balantidium polyvacuolum* in the hindgut of Xenocyprinae fishes providing new insights into the relationship among protozoa, gut microbiota and host

**DOI:** 10.3389/fmicb.2023.1295456

**Published:** 2023-11-21

**Authors:** Xialian Bu, Zhongyang Li, Weishan Zhao, Qingwen Zeng, Yushun Chen, Wenxiang Li, Hong Zou, Ming Li, Guitang Wang

**Affiliations:** ^1^Key Laboratory of Breeding Biotechnology and Sustainable Aquaculture, Institute of Hydrobiology, Chinese Academy of Sciences, Wuhan, Hubei, China; ^2^College of Advanced Agricultural Sciences, University of Chinese Academy of Sciences, Beijing, China

**Keywords:** *Balantidium polyvacuolum*, gut microbiota, short-chain fatty acids (SCFAs), protozoa, co-occurrence network

## Abstract

**Introduction:**

Parasitic ciliates are protozoans with a global distribution. Along with the gut microbiota, they have formed a micro-ecosystem that affects the host’s nutrition, metabolism, and immunity. The interactions and relationships among the three components of this microecosystem (protozoa, gut microbiota, and host) remain only partially understood. Xenocypris fish and the unique ciliate *Balantidium polyvacuolum* in its hindgut are good materials to study the interplay.

**Methods:**

In this study, 16S rRNA gene amplicon sequencing and short-chain fatty acids (SCFAs) identification were used. Network was also constructed to understand their relationships.

**Results:**

We found that the gut microbiota of *B. polyvacuolum*-infected *X. davidi* and *X. argentea* had higher diversity, richness, and evenness than uninfected ones. *B. polyvacuolum* could lead to an increase of *Fusobacterium* and Chloroflexi in both *X. davidi* and *X. argentea*, while significantly increase the abundance of genera *Romboutsia* and *Clostridium* in *X. argentea*. Besides, *B. polyvacuolum* could significantly increase the content of total SCFAs and acetic acid in *X. davidi* and increase the concentrations of propionic, isobutyric and butanoic acids in *X. argentea*. Furthermore, correlation analyses showed that *B. polyvacuolum* may alter SCFAs by affecting key SCFAs-producing bacteria such as *Clostridium* and *Cetobacterium*.

**Discussion:**

This study greatly expands our understanding of relationships among *B. polyvacuolum*, gut microbiota and host Xenocypris fish, which sheds new insights into the mechanism of interaction among protozoa, gut microbiota and host.

## 1 Introduction

Protozoa are single-celled organisms with a wide distribution, many of whom have adopted a parasitic lifestyle. These parasitic protozoa are capable of infecting a diverse array of hosts, including humans (Burgess et al., 2017), birds, reptiles (Koèíková et al., 2018), amphibians (Zhao et al., 2022a), fish (Zhao et al., 2022b), and insects (de Graaf et al., 2011). They normally live in the intestinal ecosystems, and cause a significant impact on the host (Burgess et al., 2017; Li et al., 2018). These intestinal parasites directly interact with the densely populated gut symbiotic bacteria, which may influence the composition of the gut microbiota. Evidence shows that protists from the genus Blastocystis can increase bacterial alpha diversity in the human intestine (Nieves-Ramírez et al., 2018). Colonization by the protozoan *Tritrichomonas musculis* can lead to inflammasome activation and thus contribute to the host’s protection against mucosal bacterial infections (Chudnovskiy et al., 2016). Other types of parasites, like helminths and cestodes, can also affect the diversity and structure of gut microbiota (Kreisinger et al., 2015; Brealey et al., 2022). Overall, the relationship between parasitic protozoa and gut microbiota remains incompletely understood, and further investigations are needed.

Gut microbiota plays a critical role in homeostasis by creating a mucosal barrier, offering defense against pathogens, and influencing the hosts’ metabolism, nutrition, and immunity (Leung et al., 2018; Ulusan Bagci and Caner, 2022). Most of its contribution to host physiology is related to its metabolism (Consortium, 2012). The metabolism of substrates can produce beneficial metabolites like bile acids, choline, and short-chain fatty acids (SCFAs), which are essential for the host’s health and can influence the host’s immune system (Nicholson et al., 2012). The SCFAs are produced via fermentation of amino acids or carbohydrates like glucose, starch, fiber, etc. (Horiuchi et al., 2020; Krautkramer et al., 2021; Wang et al., 2022). This fermentation can strongly expand the host’s metabolic capacity (Kaoutari et al., 2013; Krautkramer et al., 2021). Considering the cohabitation of gut microbiota and intestinal protozoa, we hypothesize that there must be some complex connection among the three components of this microecosystem (protozoa, gut microbiota, and host), which remains unknown.

*Balantidium polyvacuolum* is a single-celled organism characterized by numerous contractile vacuoles inside its body (Li et al., 2009). It has strict host specificity to Xenocyprinae fishes and mainly inhabits the mucosal folds of the hindgut (Li et al., 2009), so it’s a good material to study the interplay among protozoa, gut microbiota, and host. Up to now, there was only one report about the gut microbial communities of *X. argentea* (Yang et al., 2022). Herein, we applied the 16S rRNA gene amplicon sequencing of the gut microbiota and SCFAs identification in *X. davidi* and *X. argentea*, and then applied microbial co-occurrence network, random forest models, and correlation analysis to explore the relationships among the three components.

## 2 Materials and methods

### 2.1 Sample collection

Host fish *X. davidi* (infection *n* = 6, control *n* = 4, average weight 113.9 ± 17.4 g) and *X. argentea* (infection *n* = 6, control *n* = 3, average weight 123.6 ± 29.2 g) were collected from the Wuhan Section of the Yangtze River in Xianning City, Hubei Province, China, in July 2022. Fishes were anesthetized using 0.02% tricaine methane sulfonate (MS-222, Sigma) according to the manufacturer’s protocol and dissected in accordance with the protocols approved by the Animal Ethics Committee of Institute of Hydrobiology, Chinese Academy of Sciences (IHB/LL/2023036). The hindguts were removed under aseptic conditions. After microscopic examination to confirm the presence of *B. polyvacuolum*, the contents of the hindgut were harvested, and subsequently used for the microbiota analysis and SCFAs analysis.

### 2.2 DNA extraction and 16S rRNA gene amplicon sequencing

Intestinal microbiota DNA was extracted using the QIAamp DNA Stool Mini Kit (Qiagen, Germany) according to the manufacturer’s instructions. The quality and concentration of DNA were determined by 1.0% agarose gel electrophoresis and a NanoDrop^®^ ND-2000 spectrophotometer (Thermo Scientific Inc., USA). The DNA samples were kept at −80°C until further use. The hypervariable region V3-V4 of the bacterial 16S rRNA gene was amplified with primer pairs 338F and 806R using an ABI GeneAmp^®^ 9700 PCR thermocycler (ABI, CA, USA). Purified amplicons were pooled in equimolar amounts and paired-end sequenced on an Illumina NovaSeq PE250 platform according to the standard protocols by Majorbio Bio-Pharm Technology Co. Ltd. (Shanghai, China). The raw sequencing reads were deposited into the NCBI Sequence Read Archive (SRA) database under the BioSample number PRJNA981639.

### 2.3 Extraction and identification of SCFAs

For the SCFAs extraction, 20 mg of intestinal contents were placed into 2 mL grinding tubes and 800 μL of water containing 0.5% phosphoric acid was added. The samples were frozen and ground at 50 Hz for 3 min repeated twice, followed by ultrasonic for 10 min, and centrifugation at 4°C and 13000 × *g* for 15 min. Two hundred μL of the supernatant aqueous solution was removed into a 1.5 mL centrifuge tube. After that, 200 μL of N-butanol solvent containing internal standard 2-ethylbutyric acid (10 μg/mL) was added. After vortexing for 10 s, exposing to ultrasound at a low temperature for 10 min, and centrifuging at 4°C and 13000 × *g* for 5 min, the supernatant was carefully transferred to sample vials. The gas chromatography-mass spectrometry (GC-MS) analysis was conducted on an Agilent 8890B gas chromatography coupled to an Agilent 8890B/5977B mass selective detector with an inert electron impact (EI) ionization source and the ionization voltage of 70eV (Agilent, USA). Analyte compounds were separated with an HP-FFAP (30 m × 0.25 mm × 0.25 μm) capillary column, using 99.999% helium as a carrier gas at a constant flow rate (1 mL/min). The GC column temperature was programmed to hold at 80°C and rise to 120°C at a rate of 20°C per minute, then rise to 160°C at a rate of 5°C per minute, and finally hold at 220°C for 3 min. The injection volume of samples was 1 μL and introduced in splitting mode (10:1) with the inlet temperature of 180°C. The ion sources temperature was 230°C and the quadrupole temperature was 150°C. GC-MS was in selected-ion monitoring mode. Compounds were identified and quantified by the Masshunter software (v10.0.707.0, Agilent, USA). The mass spectrum peak area of the analyte was used as the ordinate and the concentration of the analyte as the abscissa to draw a linear regression standard curve for sample concentration calculation: the mass spectrum peak area of the sample analyte was substituted into the linear equation to calculate the concentration result.

### 2.4 Analysis of 16S rRNA gene sequences

The data were analyzed through the free online platform of majorbio choud platform.^1^ Low-quality reads (length <50 bp or quality score of <20) were removed from raw reads by fastp V.0.19.6 (Chen et al., 2018). The clean data were then assembled using FLASH V.1.2.11 (Magoè and Salzberg, 2011). Then, the optimized sequences were clustered into operational taxonomic units (OTUs) using UPARSE V.7.1 (Edgar, 2013) at a 97% sequence similarity level. The most abundant sequence for each OTU was selected as a representative sequence. The taxonomy of each OTU representative sequence was analyzed by RDP Classifier V.2.2 (Wang et al., 2007) against the Silva database V.123 using a confidence threshold of 0.7.

The following analysis and visualization were performed in RStudio V.4.1.1 (R Core Team, 2019). First, the relative abundance of microbial taxa was compared among different groups at phylum and genus levels. Then, the α diversity indices including Richness, ACE, and Shannon were calculated. For the β diversity analysis, non-metric multi-dimensional scale analysis (NMDS) based on the Bray-Curtis distance algorithm also was used to show the distribution and relationship among samples. Linear discriminant analysis coupled with effect size (LEfSe) was used to find species with significant differences at the genus level. Meanwhile, random forest analysis (RFA) was used to classify the most discriminant genera. Finally, Spearman’s correlation between the discriminant genera of infected and uninfected groups selected by RFA was analyzed.

### 2.5 Construction of the microbial network

The Molecular Ecological Network Analysis (MENA) pipeline^2^ was used to construct networks as previously described (Deng et al., 2012). First, only the top 100 dominant OTUs in each group were used for the network construction. Second, the Pearson correlation was calculated based on the log-transformed OTU abundances and then only relationships with a correlation coefficient *r* ≥ 0.2 and *p*-value < 0.05 were used to construct networks.

Global network indexes, including the average clustering coefficient, average path length, connectivity, and modularity were calculated as described previously (Deng et al., 2012). The topological roles of nodes in networks were evaluated and divided into four types based on the within-module connectivity (Zi) and among-module connectivity (Pi), which were network hubs (*Z*i > 2.5, *P*i > 0.62), module hubs (*Z*i > 2.5, *P*i ≤ 0.62), connectors (*Z*i ≤ 2.5, *P*i > 0.62), and peripheral nodes (*Z*i ≤ 2.5, *P*i ≤ 0.62). Network hubs, module hubs, and connectors can be seen as key nodes in network structuring (Olesen et al., 2007). Networks were visualized by Gephi software V0.10 (Bastian et al., 2009).

### 2.6 Analysis of SCFAs

The Shapiro and Bartlett tests were used to test the normality and homogeneity of variances, respectively. Then appropriate testing methods were chosen for the inter-group difference testing. Results were visualized with the ggplot2 package (Villanueva and Chen, 2019) in R (R Core Team, 2019).

### 2.7 Correlation analysis of microbiota and SCFAs

First, we used only the discriminant genera selected by RFA for the network construction. Second, Spearman’s correlation coefficient was calculated and only relationships with the *p*-value ≤ 0.05 were kept for the following analysis. Then, node properties were calculated. The correlation networks were visualized by the igraph package (Csardi and Nepusz, 2006) in R (R Core Team, 2019).

## 3 Results

### 3.1 Impact of *B. polyvacuolum* on gut microbiota

**FIGURE 1:**
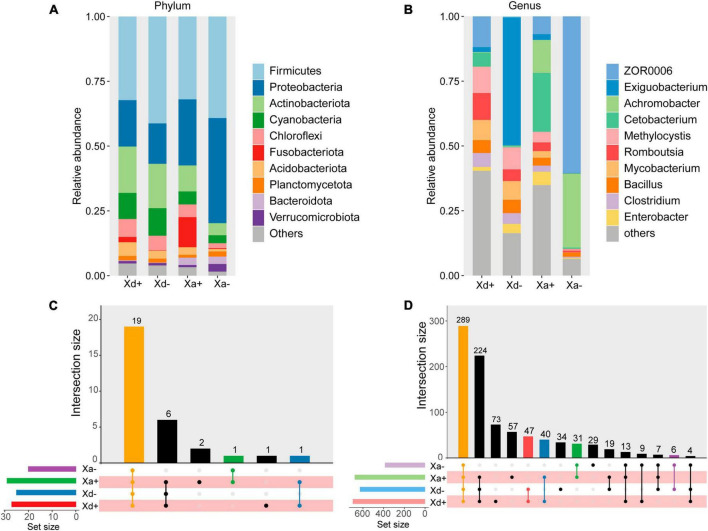
The relative abundance of microbial taxa at phylum and genus levels and the species composition of each group. **(A)** *X. davidi* infected with *B. polyvacuolum* (Xd+) and without *B. polyvacuolum* (Xd-). “Others” comprises the sum of different taxa, aside from the top 10 most abundant categories. **(B)** *X. argentea* infected with *B. polyvacuolum* (Xa+) and without *B. polyvacuolum* (Xa-). “Others” comprises unclassified genera in the top eighteen abundant categories. Upset diagrams showing the species composition in four groups at the phylum **(C)** and genus **(D)** levels. The data for drawing can be found in Supplementary Tables 1–4.

At the phylum level, Firmicutes, Proteobacteria, Actinobacteriota, Cyanobacteria and Chloroflexi were the top five relatively abundant taxa in both *B. polyvacuolum*-infected *X. davidi* (Xd+) and uninfected *X. davidi* (Xd-). In the *B. polyvacuolum*-infected *X. argentea* (Xa+), Firmicutes, Proteobacteria, Fusobacteriota, Actinobacteriota, and Chloroflexi were the top five relatively abundant taxa, while Firmicutes and Proteobacteria took the large part in the uninfected *X. argentea* (Xa-) (Figure 1A). The relative abundance of Chloroflexi was significantly higher (*p* = 0.034) in Xa+ than in Xa-, while Fusobacteriota was higher in both Xd+ and Xa+. Regardless of the *B. polyvacuolum* infections, the relative abundance of Proteobacteria was higher in *X. argentea* compared to *X. davidi*.

At the genus level, ZOR0006, *Methylocystis*, *Romboutsia*, and *Mycobacterium* were the top four relatively abundant taxa in Xd+ , while *Exiguobacterium* was abundant in Xd-. In Xa+, *Cetobacterium*, *Achromobacter*, and ZOR0006 were the top three relatively abundant taxa, while ZOR0006 and *Achromobacter* were abundant in Xa- (Figure 1B). The abundance of *Romboutsia* and *Clostridium* was significantly higher in Xa+ than in Xa- (*p* = 0.048 and 0.033, respectively), while the level of ZOR006 was significantly higher in Xa- (*p* = 0.024). The abundance of *Exiguobacterium* was significantly higher in Xd- than in Xd+ (*p* = 0.019). The four groups had 19 phyla and 289 genera in common (Figures 1C, D). The number of genera shared by Xd+ and Xa+ was 40. The total numbers of genera identified in groups Xd+, Xd-, Xa+, and Xa- were 458, 395, 436, and 364, respectively.

**FIGURE 2:**
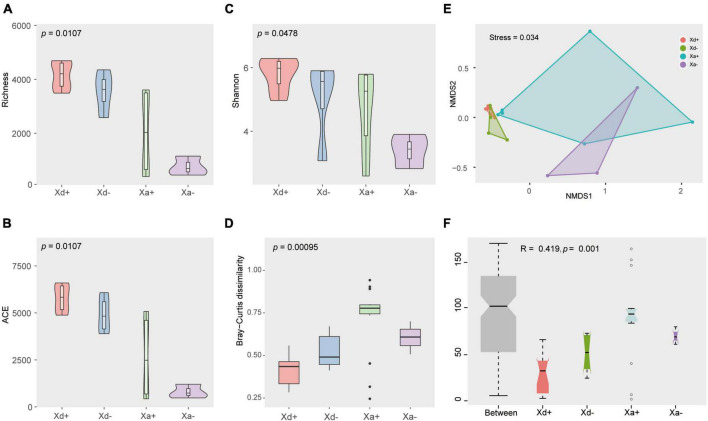
The structural features of the gut microbiota in four groups. Richness **(A)**, ACE **(B)**, and Shannon **(C)** indices of the alpha diversity. **(D)** Beta diversity. **(E)** Non-metric multi-dimensional scale analysis (NMDS) showing the distribution and relationship among samples. Dots represent samples. **(F)** ANOSIM results. *p* < 0.05 indicates a significant difference. The data for drawing can be found in Supplementary Tables 5–7.

As for the alpha diversity, richness, ACE, and Shannon indices were calculated (Figures 2A–C). There were no significant differences between Xd+ and Xd- or between Xa+ and Xa-. Same for the beta diversity (Figure 2D). NMDS results showed that samples from different groups were scattered in different regions, except for partial overlaps (Figure 2E). Most of these overlaps occurred between the same species of fish (Xd+ /Xd- or Xa+ /Xa-). The ANOSM results also showed significant differences between groups (Figure 2F).

**FIGURE 3:**
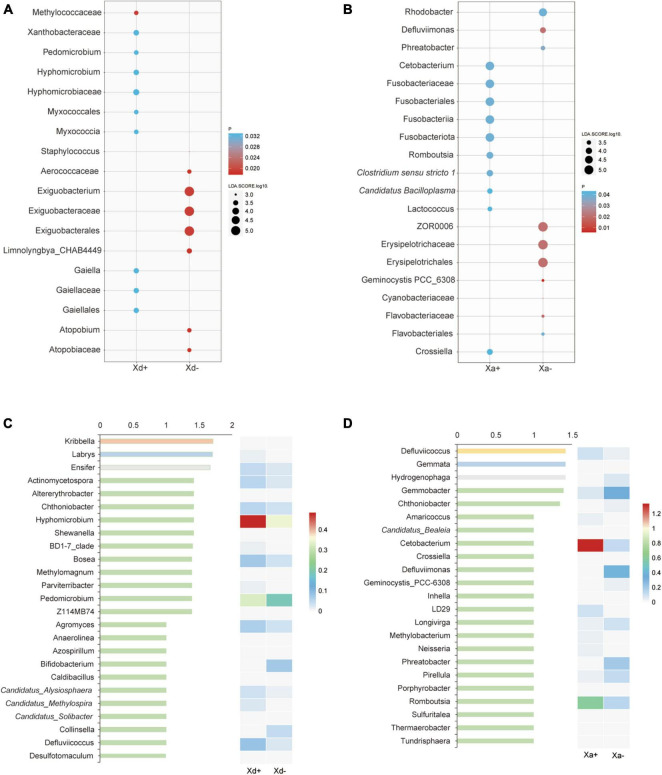
The most discriminant genera of uninfected and infected groups selected by LEfSe **(A,B)** and the random forest model **(C,D)**. **(A,C)** panels show groups Xd+ and Xd-, whereas **(B,D)** show groups Xa+ and Xa-. The *x*-axis of the bar plot represents IncMSE (increase in mean squared error) and heat map based on the relative abundance of genera in **(C,D)**. The data for drawing can be found in Supplementary Tables 8, 9.

**FIGURE 4:**
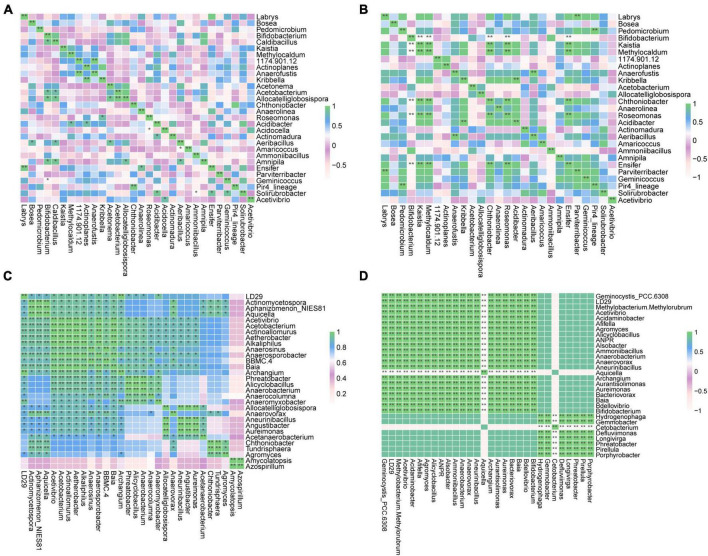
Heatmap of the Spearman’s correlation between the discriminant genera of uninfected and infected groups selected by a random forest model (*FDR < 0.05, **FDR < 0.01). **(A)** Group Xd+ . **(B)** Group Xd-. **(C)** Group Xa+ . **(D)** Group Xa-. The data for drawing can be found in Supplementary Table 10.

Genera exhibiting significant differences among groups were screened by the two different methods LEfSe and RFA. The LEfSe analysis found that 18 taxa (Xd+ vs. Xd-) and 20 taxa (Xa+ vs. Xa-) showed significantly differences (Figures 3A, B). Meanwhile, the RFA showed that the significantly different taxa were 25 (Xd+ vs. Xd-) and 23 (Xa+ vs. Xa-), respectively (Figures 3C, D). Both analyses identified *Hyphomicrobium*, *Methylomagnum*, *Pedomicrobium*, *Caldibacillus*, and *Collinsella* (Xd+ vs. Xd-), and *Crossiella*, *Defluviimonas*, *Geminocystis*, and *Phreatobacter* (Xa+ vs. Xa-) as significantly different genera. Compared to respective control group, most of the genus were negatively correlated in Xd+ (Figures 4A, B), while positively corelated in Xa+ (Figures 4C, D).

### 3.2 The co-abundance network of the gut microbiota

**FIGURE 5:**
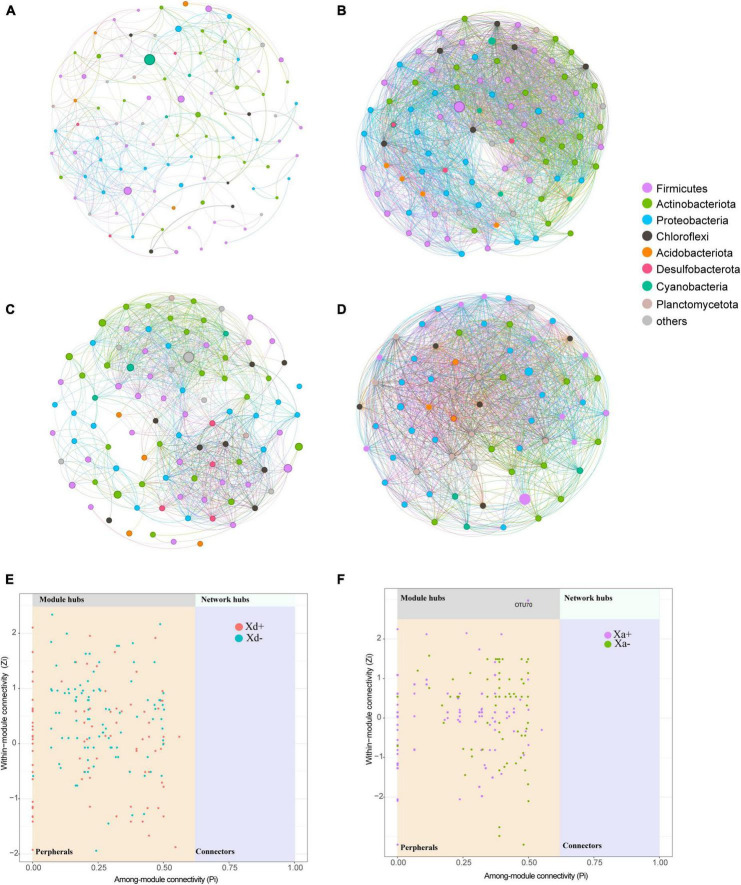
Co-occurrence networks based on the top 100 dominant genera. Node size represents the node degree and color represents the phylum. **(A)** Network of group Xd+ . **(B)** Network of group Xd-. **(C)** Network of group Xa+ . **(D)** Network of group Xa-. **(E)** Classification of nodes in Xd+ and Xd- networks. **(F)** Classification of nodes in Xa+ and Xa- networks. The data for drawing can be found in Supplementary Table 11.

We sought to determine the co-occurrence patterns of the microbiota in the four groups. Results showed significant differences in network properties among groups (Table 1 and Figure 5). Xd+ has higher modularity and fewer network edges, compared with Xd-. Its network nodes mainly consisted of Firmicutes, Actinobacteriota, and Proteobacteria (Figure 5A), and the largest node belonged to Actinobacteriota in Xd+ , and to Firmicutes in Xd- (Figures 5A, B). As for Xa+ , it had more network nodes and higher modularity than Xa- (Figures 5C, D), and the main network nodes of it were Firmicutes, Actinobacteriota, and Proteobacteria (Figure 5C). Only one module hub was detected in Xa+ and no connectors were detected in the four groups (Figures 5E, F).

**TABLE 1:** Major network topological properties in the four groups.

Group	Nodes	Edges	Avg. path length	Clustering coefficient	Modularity
Xd+	99	401	2.94	0.53	0.50
Xd-	100	2493	1.50	0.76	0.28
Xa+	97	1114	2.10	0.74	0.39
Xa-	70	1642	1.32	0.83	0.17

### 3.3 Distribution of SCFAs in the infected and uninfected groups

**FIGURE 6:**
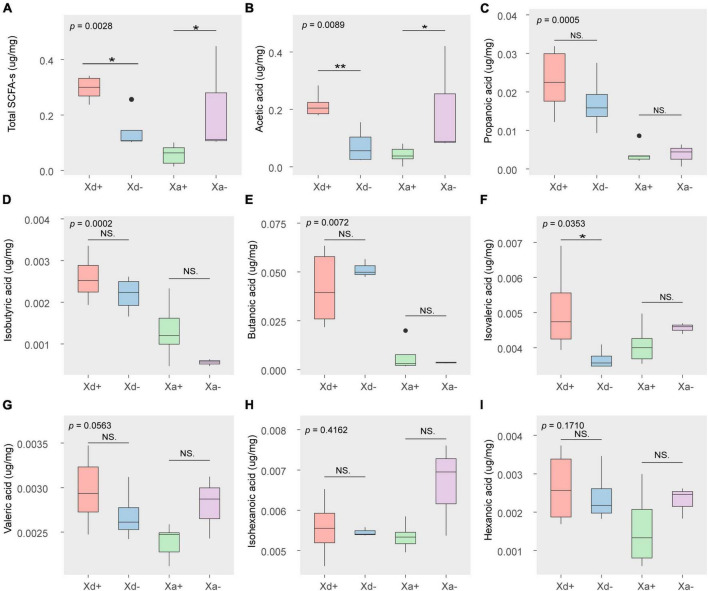
The concentration of SCFAs in different groups (μg/mg wet weight). **(A)** the total amount of SCFAs. The amount of acetic acid **(B)**, propanoic acid **(C)**, isobutyric acid **(D)**, butanoic acid **(E)**, isovaleric acid **(F)**, valeric acid **(G)**, isohexanoic acid **(H)**, and hexanoic acid **(I)**. The data for drawing can be found in Supplementary Table 12

The content of acetic acid was the highest among the eight SFCAs, followed by propanoic acid and butanoic acid (Figure 6). The total SCFAs, acetic acid and isovaleric acid significantly differed between Xd+ and Xd- (Figures 6A, B, F), while the total SCFAs and acetic acid also showed significant differences between Xa+ and Xa- (Figures 6A, B).

### 3.4 The correlation between the SCFAs and most discriminant genera in uninfected and infected groups

**FIGURE 7:**
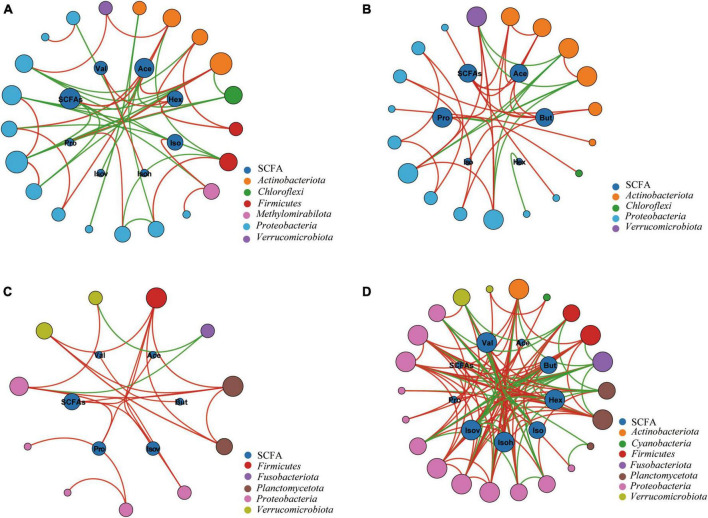
Networks between the SCFAs and the most highly discriminant genera selected by the RFA analysis. Node size is proportional to node connectivity. Outer nodes in different colors represent different phyla and inner nodes represent different SCFAs. Red lines indicate positive interactions and green lines indicate negative interactions. **(A)** Group Xd+ . **(B)** Group Xd-. **(C)** Group Xa+ . **(D)** Group Xa-. The data for drawing can be found in Supplementary Table 13

In order to explore the relationship between the intestinal microbiota and SCFAs, we conducted a network analysis. It indicated a significant correlation between gut microbiota and SCFAs (Figure 7). Proteobacteria and Actinobacteria dominated the large modules in the networks of Xd+ and Xd- (Figures 7A, B). The total SCFAs and acetic acid in Xd+ had a significantly positive correlation with *Collinsella* (Figure 7A). Besides, more SCFAs in Xd+ significantly correlated with gut microbiota than that in Xd-. The Xd+ network had more connectors than the Xd- network (Figures 7A, B). Proteobacteria was the only dominant module in both Xa+ and Xa- (Figures 7C, D). Network nodes in Xa+ was fewer than that in Xa-, and most correlations in Xa+ were positive (Figure 7C).

## 4 Discussion

### 4.1 *B. polyvacuolum* affects the composition of gut microbiota in *X. davidi* and *X. argentea*

In this study, we used *X. davidi* and *X. argentea* to analyze in detail the gut microbial composition and structure in response to infection with *B. polyvacuolum*. Results showed that Firmicutes, Proteobacteria, and Actinobacteriota were the predominant microbial phyla in *X. davidi* and *X. argentea*’s hindgut, which corresponds to previous reports in other fish species (Wu et al., 2012; Baldo et al., 2015; Belkova et al., 2017). Infection with *B. polyvacuolum* can cause an increase in the overall diversity of gut microbiota, as well as an increase in the relative abundance of Fusobacteria and Chloroflexi, in both *X. davidi* and *X. argentea*. Previous studies showed that Fusobacteria and *Cetobacterium* were the dominant phylum and genus, respectively, in the fish gut (Roeselers et al., 2011; Liu et al., 2016). Chloroflexi was also found in domesticated zebrafish and in the Nile tilapia (*Oreochromis niloticus*) (Pham et al., 2008; Bereded et al., 2020). The diversity and relative abundance of Fusobacteria was positively correlated with antibody production and resistance to infections (Knutie et al., 2017), it can be inferred that *X. davidi* and *X. argentea* with *B. polyvacuolum* may be healthier than the uninfected ones.

We also observed a significant increase of genera *Romboutsia* and *Clostridium* in *B. polyvacuolum*-infected *X. argentea*. *Romboutsia* plays an important role in carbohydrate utilization, single amino acids fermentation, and anaerobic respiration (Gerritsen et al., 2019). This genera was proved to be closely related to numerous species of *Clostridium* (Wang et al., 2015). *Clostridium* is commonly abundant in the gut of herbivorous and omnivorous fish, which has the ability to degrade cellulose (Liu et al., 2016; Yukgehnaish et al., 2020). Considering that *X. davidi* and *X. argentea* mainly feed on plant debris and algae (Sifa, 2000), it can be inferred that *Clostridium* in the hindgut of *X. davidi* and *X. argentea* also plays an important role in degrading cellulose.

In addition, we also found that among groups without *B. polyvacuolum* infection, ZOR006 and *Exiguobacterium* were dominant genera in *X. davidi* and *X. argentea*, respectively. Meanwhile, the alpha diversity showed that the gut microbiota of *B. polyvacuolum*-infected *X. davidi* and *X. argentea* had higher diversity, richness, and evenness than the uninfected ones. It may indicate that *B. polyvacuolum* had a beneficial effect on the balance of gut microbiota. Higher microbial diversity levels and more even communities could be signs of good gut health, which could be beneficial for the productivity of aquaculture (Infante-Villamil et al., 2021). It can be inferred that *B. polyvacuolum* contributed to the richness and evenness of the gut microbial community and made the gut microenvironment more diverse and stable. We also observed higher modularity in the *B. polyvacuolum*-infected *X. davidi*. Modularity is important for the stability of ecological networks (Olesen et al., 2007). Higher modularity indicated that the microbial ecosystem was more stable in *B. polyvacuolum*-infected *X. davidi.*

### 4.2 *B. polyvacuolum* may affect SCFAs synthesis in *X. davidi* and *X. argentea* through gut microbiota

Sequencing and short-chain fatty acids are the end products of gut microbial fermentation, mainly concentrated in the hindgut of aquatic animals (Clements et al., 1994). Gut microbial-derived SCFAs mainly include acetic, propionic, butyric, valeric, and caproic acids, and the first three are commonly the most abundant SCFAs (Trachsel et al., 2016). On one hand, the synthesis of SCFAs is correlated with microbial composition and environmental conditions (Louis et al., 2014), though it can also be affected by the species of fish. Normally, carnivorous fish have relatively higher SCFA concentrations than herbivorous and omnivorous species (Clements et al., 2014).

With *B. polyvacuolum* infection, the abundance of total SCFAs, acetic acid, and isovaleric acid in *X. davidi* and propionic, isobutyric and butanoic acids in *X. argentea* were significantly increased. In other words, the presence of *B. polyvacuolum* could lead to an increase in SFCAs, as the increased SCFAs may be a direct product of the increased diversity of gut microbiota. Research has shown that propionic acid could decrease the levels of fatty acids in liver and plasma, suppress appetite, and exhibit immunosuppressive effects (Sa’ad et al., 2010). Considering the crucial role of SCFAs in maintaining gut homoeostasis and monitoring the relationship with the host, its immune system and intestinal commensal microbes (Trachsel et al., 2016; Tran et al., 2020), the higher total SCFAs may indicate improved gut health in the *B. polyvacuolum*-infected *X. davidi* and *X. argentea*.

The high concentration of SCFAs in the *B. polyvacuolum*-infected *X. davidi* and *X. argentea* may be related to the high abundance of gut microbiota. In other words, there was a positive relationship between the diversity and composition of gut microbiota and the production of SCFAs. For instance, the abundance of *Clostridium* was significantly higher in the *B. polyvacuolum*-infected *X. argentea*. *Clostridium* is considered to be a probiotic and the main acetic acid-producing bacterium (Guo et al., 2019; Xu et al., 2020). It can be used by colonocytes to generate energy, maintain gut anaerobic conditions, preserve gut barrier integrity, and limit pro-inflammatory cytokines (Singh et al., 2023). It was reported that *Clostridium butyricum* can modulate intestinal metabolic capacities and improve the intestinal SCFA content in *Marsupenaeus japonicus* (Duan et al., 2018). It can also enhance the production of butyric acid and propanoic acid, as well as the activity of catalase and lysozyme in the intestine of common carp (Meng et al., 2021).

We also found an increased abundance of *Cetobacterium* in the *B. polyvacuolum*-infected *X. davidi* and *X. argentea*. *Cetobacterium* is also a good SCFAs producer and its metabolites, such as acetic, propionic, and butanoic acid, can improve fish health (Bhute et al., 2020; Wang et al., 2021; Xie et al., 2021), so the increased *Cetobacterium* may be the sign that *B. polyvacuolum*-infected *X. davidi* and *X. argentea* are in good health. Bacteria from the genus *Collinsella* were also correlated with the total SCFAs and acetic acid in *X. davidi*. This genus is also proved to be an effective SCFA producer (Yin et al., 2013). All these findings showed that *B. polyvacuolum* can regulate the levels of SCFAs by altering the gut microbiota composition.

## 5 Conclusion

This study revealed the interactions among parasitic protozoa, gut microbiota, and host metabolism. The results showed that *B. polyvacuolum* can affect the abundance and structure of gut microbiota in *X. davidi* and *X. argentea*. It increased the diversity, richness, and evenness of gut microbiota and enhanced its stability, which could be the sign that host with *B. polyvacuolum* are in good health. Meanwhile, SCFAs producers like *Cetobacterium* and *Clostridium* were also increased in *B. polyvacuolum*-infected *X. davidi* and *X. argentea*. Accordingly, the abundance of total SCFAs, acetic acid, and isovaleric acid significantly increased in *B. polyvacuolum*-infected *X. davidi*, while the concentrations of propionic, isobutyric and butanoic acids also increased in the *B. polyvacuolum*-infected *X. argentea*. These findings may indicate that *B. polyvacuolum* were a commensal member of the gut. Our findings provided valuable insights into the relationships between parasitic ciliates, gut microbiota, and host metabolism. This study may deepen the understanding of the impact of parasitic protozoa on host and gut microbiota.

## Data availability statement

The datasets presented in this study can be found in online repositories. The names of the repository/repositories and accession number(s) can be found below: https://www.ncbi.nlm.nih.gov/bioproject/PRJNA981639/.

## Ethics statement

The animal studies were approved by the Animal Ethics Committee of Institute of Hydrobiology, Chinese Academy of Sciences. The studies were conducted in accordance with the local legislation and institutional requirements. Written informed consent was obtained from the owners for the participation of their animals in this study.

## Author contributions

XB: Formal analysis, Writing – original draft. ZL: Software, Visualization, Writing – review and editing. WZ: Funding acquisition, Software, Writing – review and editing. QZ: Software, Writing – original draft. YC: Writing – review and editing. WL: Writing – review and editing. HZ: Writing – review and editing. ML: Funding acquisition, Supervision, Writing – review and editing. GW: Funding acquisition, Supervision, Writing – review and editing.

## Footnotes

1
cloud.majorbio.com

2
http://ieg2.ou.edu/MENA/
